# Cognitive Empathy in Subtypes of Antisocial Individuals

**DOI:** 10.3389/fpsyt.2021.677975

**Published:** 2021-07-05

**Authors:** Shou-An A. Chang, Scott Tillem, Callie Benson-Williams, Arielle Baskin-Sommers

**Affiliations:** ^1^Department of Psychology, Yale University, New Haven, CT, United States; ^2^Department of Psychology, University of Michigan, Ann Arbor, MI, United States

**Keywords:** cognitive empathy, antisocial, psychopathy, callous-unemotional, theory of mind, perspective-taking

## Abstract

Cognitive empathy allows individuals to recognize and infer how others think and feel in social situations and provides a foundation for the formation and maintenance of mutually constructive relationships. It may seem intuitive to assume that individuals who engage in antisocial behavior, who disregard the rights of others, might have problems with cognitive empathy. However, careful examination of the literature suggests that any dysfunction in cognitive empathy associated with antisociality varies by subtype of antisocial individual and is specific to subcomponents of cognitive empathy. In this review, we (1) briefly define subtypes of antisocial individuals (“psychopathic” vs. “antisocial-only”), (2) summarize specific components of cognitive empathy; (3) review existing literature examining cognitive empathy through questionnaires, behavioral tasks, and neuroimaging within different antisocial subtypes; and (4) discuss the limitations of the current research and potential future directions. Individuals in the psychopathic subtype fail to implicitly engage in cognitive empathy, and potentially lack insight into this issue reflected in no self-reported problems with cognitive empathy, but show an ability to engage in cognitive empathy when explicitly required. Individuals in the antisocial-only subtype appear able to engage in cognitive empathy, showing no differences on questionnaire or behavioral tasks that tap explicit cognitive empathy, but may display subtle difficulties accurately inferring (affective theory of mind) the emotions of others. We end the review by noting areas for future research, including the need to: (1) document the patterns of equifinality that exist across levels of analysis for these antisocial subtypes; (2) examine the temporality of empathy and antisociality development; (3) carefully consider and label subcomponents of cognitive empathy in research on antisocial behavior; and (4) investigate the intersection among environmental experiences, cognitive empathy, and antisocial behavior.

Successful social interaction requires the ability to represent what other people are thinking and feeling. This ability, often referred to as cognitive empathy, helps individuals predict and interpret others' behaviors, develop meaningful social relationships, communicate effectively, and engage in appropriate moral reasoning (1, 2). Cognitive empathy is critical in everyday social interactions, and a variety of psychiatric disorders, including autism, bipolar disorder, and schizophrenia (3–5) are characterized by difficulties with cognitive empathy. However, psychiatric disorders associated with antisocial behaviors, which are actions that violate social norms (e.g., lying, intimidation, inflicting physical harm), show mixed effects with regard to cognitive empathy dysfunctions.

It seems intuitive to think that the actions of those who continually violate the rights of others are, in part, a reflection of the person's difficulty in representing and understanding what others might be thinking or feeling (6, 7). However, careful examination of the empirical work on cognitive empathy abilities in antisocial individuals indicates that the relationship between cognitive empathy and antisociality is far more complex than this intuitive account. The primary goal of this paper is to review research on cognitive empathy in subtypes of antisocial individuals. To this end, we (1) briefly describe two subtypes of individuals who engage in chronic and damaging antisocial behavior, (2) summarize the specific components of cognitive empathy that will be examined in this paper; (3) review existing literature examining cognitive empathy within different antisocial subtypes; and (4) discuss the limitations of the current research and potential future directions.

## Subtypes of Antisocial Individuals: the Psychopathic Vs. Antisocial-Only Subtype

**Figure 1:**
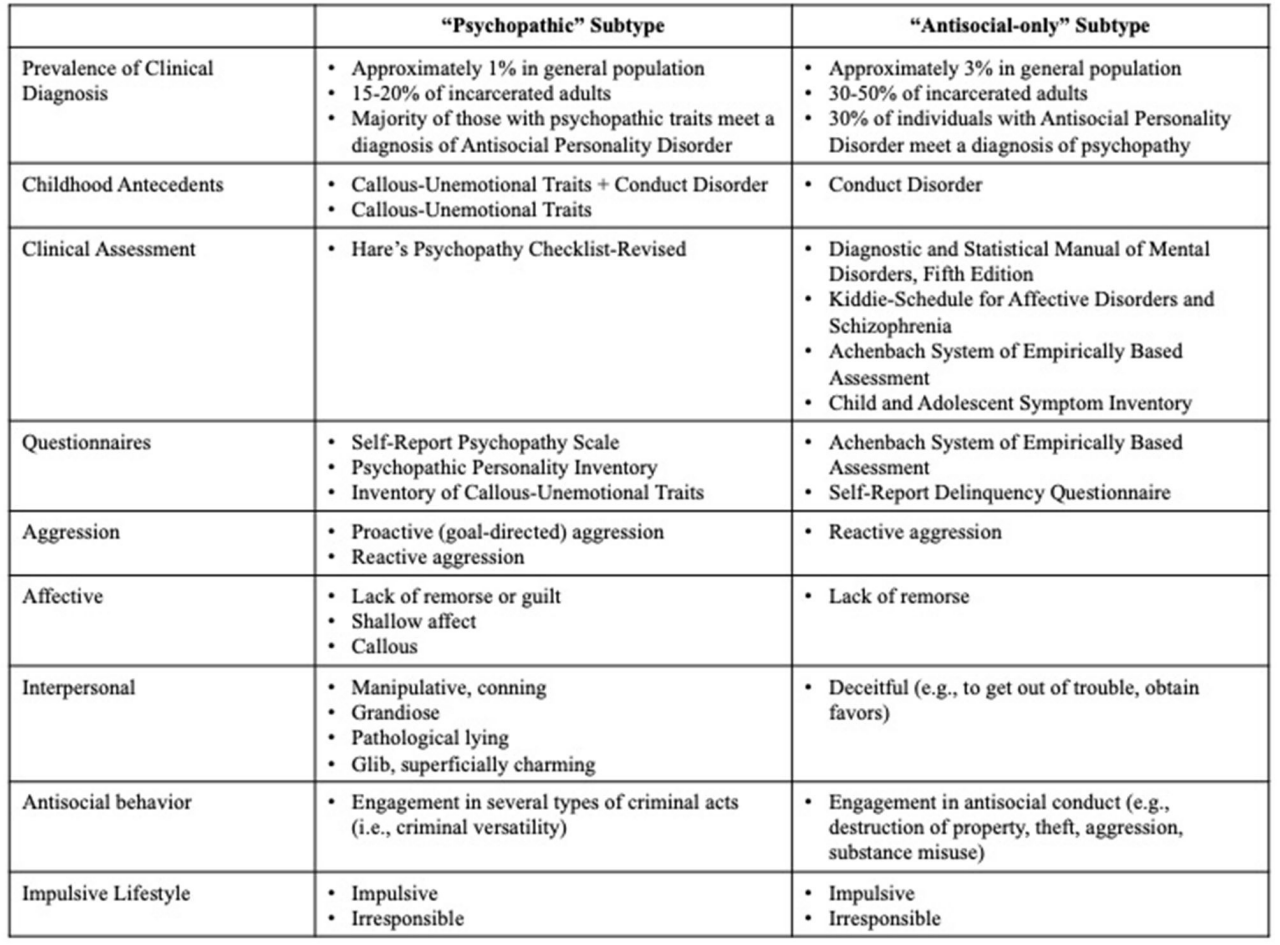
Clinical assessment tools and phenotypes for “psychopathic” subtype vs “antisocial-only” subtype. Information represents common tools and tendencies across subtypes of antisocial individuals.

Individuals chronically engaging in antisocial behaviors are at risk for a variety of adverse life outcomes, such as suicide, school dropout, unemployment, psychopathology, substance abuse, and incarceration (8, 9). Moreover, estimates of the financial impact of antisocial behavior (e.g., the cost of law enforcement, incarceration, property damage, loss of wages, healthcare, etc.) on society exceed $2 trillion annually in the United States alone (10). Research demonstrates that there are two clinically meaningful subtypes of individuals engaging in high levels of antisocial behavior (see Figure 1) (11–14).

The first subtype, which we term the “psychopathic” subtype, are individuals infamous for their prolific antisocial behavior and their ability to be interpersonally manipulative and charming. They engage in elaborate cons, callously assault others, impulsively look for adventures, and chronically commit antisocial acts in order to obtain their goals (e.g., money, power, thrills). Psychopathic individuals commit two to three times more violent and non-violent crimes than non-psychopathic individuals, recidivate at a much higher rate, and are responsible for a disproportionate share of the estimated annual costs associated with crime in the United States (10). In his seminal writings, Cleckley states that the individual with psychopathy “. cannot be depended upon to show the ordinary responsiveness to special consideration or kindness or trust. No matter how well he is treated… he shows no consistent reaction of appreciation except superficial and transparent protestations. Such gestures are exhibited most frequently when he feels they will facilitate some personal aim” [(15), p. 354]. The individual with psychopathy, therefore, uses their ability to connect interpersonally and emotionally at a surface level in order to arrange their relationships and social transactions in ways that will benefit them, usually at the expense of others.

For adults, in both clinical and research settings, the gold standard assessment of psychopathy is Hare's Psychopathy Checklist-Revised Revised [PCL-R (16)], an interview-based measure of the interpersonal (charm, gradisotiy), affective (shallow affect, lack of empathy, lack of remorse), impulsive (poor behavioral control, irresponsibility), and antisocial (engagement in criminal activity, aggression) subcomponent characteristics of this disorder. The PCL-R rates individuals on 20 different items that cut across these four characteristics on a scale from 0 to 2 for each item. In the United States, individuals with a score of 30 or above are diagnosed with psychopathy. Approximately 15–25% of incarcerated adult offenders, and 1% of the general population, meet a diagnosis of psychopathy (16–18). Other than formal diagnostic measures, some researchers utilize self-report questionnaires, such as the Self-Report Psychopathy Scale (19) or the Psychopathic Personality Inventory (20) to assess psychopathy. Though there is evidence that individuals in the psychopathic subtype engage in impression management/dissimulation (21), self-report questionnaires in a research setting are valid and reliable metrics of psychopathy and correlate well with diagnostic measures (e.g., PCL-R) in community and incarcerated samples.

Moreover, there is a growing body of research demonstrating that the interpersonal, affective, and behavioral characteristics of psychopathy emerge during childhood and often persist throughout development (22–24). Callous-unemotional (CU) traits are a specifier of conduct disorder (CD) in the Diagnostic and Statistical Manual of Mental Disorders, Fifth Edition (DSM-5) called “limited prosocial emotions,” and include callous use of others, a lack of remorse or guilt, and an absence of empathy. Researchers theorize that, in youth, the presence of CU traits, grandiose narcissism and impulsive-antisocial traits, increase risk of developing psychopathy (14, 25–30). On average, CU traits are present in 9–25% of youth offenders (25–27). In addition to conceptualizing CU traits as a qualifier of a unique subgroup of youth who also show conduct problems, some researchers examine CU traits by themselves, without consideration for conduct disorder/problems. CU traits, themselves, are predictive of antisocial behavior, academic underachievement, and interpersonal problems in some youth (31, 32). Measuring CU traits without consideration of conduct disorder/problems, effectively captures the interpersonal-affective characteristics, such as shallow affect, callousness, and a lack of empathy, of the psychopathic subtype. In addition to the diagnostic criteria provided in the DSM-5, CU traits can be assessed using self-report questionnaires or other (e.g., teacher, parent)-report questionnaires [e.g., Inventory of Callous-Unemotional Traits (33)].

The second subtype, the “antisocial-only” subtype, is defined by their chronic impulsive, irresponsible, reactively aggressive, and antisocial behavior. Unlike, the “psychopathic” subtype, these individuals are not characterized by grandiose charm and a callous, lack of empathy. Rather, individuals in this subtype are typically assessed using diagnostic criteria that reflect various antisocial acts only. Adults in this subtype can be identified diagnostically by assessing for antisocial personality disorder (ASPD) using the criteria put forth by the DSM-5. ASPD is related to repeated social norm violations, impulsivity, irresponsibility, and aggression that began in childhood to persist into adulthood (34). In order to receive a diagnosis of ASPD, individuals must meet criteria for CD prior to the age of 16 (which can be diagnosed retrospectively). In the DSM-5, youth with CD are characterized by a pattern of behaviors that violate the rights of others or societal norms in several ways (e.g., aggression to people or animals, destruction of property, theft, rule violations, etc.). In terms of prevalence, estimates suggest that between 50 and 66% of male prisoners meet criteria for ASPD (35, 36). Finally, some researchers, particularly using young samples, examine cumulative scores of conduct problems that cut across rule-breaking and aggressive behavior.

Both subtypes of individuals are known to act on impulse, display aggression, and engage in antisocial behaviors. One distinguishing aspect of the behavior of the “psychopathic” subtype is the presence of traits that reflect superficial interpersonal connections and blunted affect that impede their ability to form and maintain, meaningful, long-term relationships. On the one hand, the “psychopathic” individual draws you in with charm and manipulation, but also engages in hostile, impulsive and irresponsible behavior with an uncanny selfish drive. On the other hand, the “antisocial-only” individual engages in hostile, impulsive, and irresponsible behavior with a tinge of reactivity and brute force. Thus, despite many similarities in the actions of these individuals, a growing body of research suggests that relatively distinct socio-affective processes characterize these subtypes of individuals (11–14, 37–41). Accordingly, a closer examination of socio-affective processing could tell us *why* a particular individual continues to engage in these behaviors despite the persistence of social and legal problems. In this review, we focus on cognitive empathy as a set of socio-affective processes purportedly implicated in antisocial behavior^1^.

## Brief Review on the Measurement of Cognitive Empathy

Cognitive empathy is involved in assessing another agent's emotions, beliefs, goals, or intentions within a given situational context. It comprises of several subcomponent processes, such as perspective-taking and attributing feelings and thoughts to self and others (42, 43). More specifically, some researchers separate the ability to recognize another agent's feelings or thoughts (perspective-taking) from forming an inference about the feelings and thoughts of the other agent (sometimes called cognitive empathy, Theory of Mind (ToM), or “mentalizing”). Further, researchers often distinguish affective perspective-taking/ToM and cognitive perspective-taking/ToM. Affective perspective-taking refers to the capacity to recognize the emotional state of another agent, whereas cognitive perspective-taking reflects that ability to infer the thoughts of another agent. For example, affective perspective-taking would be when a person is able to label that, while they are happy getting invited to a party, their friend is sad about not getting invited to the party. Cognitive perspective-taking would be when a person recognizes that a co-worker does not know about the change in protocol announced at a staff meeting because the co-worker did not attend the staff meeting.

These cognitive empathy capabilities can be measured through questionnaires or experimental tasks. Several different questionnaires exist for assessing cognitive empathy. One of the most widely used questionnaires is the Interpersonal Reactivity Index [IRI (44)]. A subscale of this measure taps perspective-taking (e.g., “I try to look at everybody's side of a disagreement before I make a decision.”; “Before criticizing somebody, I try to imagine how I would feel if I were in their place.”). While questionnaire-based measures might provide broadband assessments of cognitive empathy, there is some question about the precision with which questionnaire measures, such as the IRI, specifically assess cognitive empathetic processes. For example, the perspective-taking subscale of the IRI includes some questions that are more cognitive in nature and some that reflect emotions, making it difficult to completely disentangle cognitive and affective perspective-taking. Therefore, questionnaire-based measures broadly evaluate some aspects of cognitive empathy, however, the specific subcomponent process is less clear.

Additionally, cognitive empathy can be evaluated using experimental tasks. During cognitive empathy tasks, participants are presented with scenarios or scenes, and are asked to use and integrate information about the situational context of a scene and/or the agent's actions to evaluate the agent's feelings or thoughts (e.g., “Character A just told Character B s/he could not have a piece of candy; how does Character B feel?”).

Cognitive empathy can be assessed explicitly or implicitly. Tasks explicitly evaluating cognitive empathy typically expose participants to a scenario (either by having them read a vignette, view a cartoon image or photograph, or watch a film clip). For affective perspective-taking/ToM tasks, the instructions would ask participants about different characters' feelings [e.g., “Pick which of four words best describes what the person in the photo is feeling.” (45)]. Though there is an emotion recognition component to many of these tasks, the specific question being asked in these tasks relates to representing/understanding or inferring other's emotion (not necessarily resonating with or responding to the emotions, which would fit more with the conceptualization affective/emotional empathy not covered in this review). For a cognitive ToM task, similar stimuli could be used to ask participants about the characters' beliefs, goals, or intentions [e.g., using a Sally-Anne-type false belief task (46, 47)].

In contrast, tasks implicitly evaluating components of cognitive empathy assess the degree to which an individual automatically (e.g., without instruction, unintentionally, unconsciously) assesses another agent's feelings, beliefs, goals, or intentions (48), sometimes even during an unrelated task [e.g., see (49)]. For example, using a Sally-Anne false belief task, researchers can examine the extent to which a participant infers, or anticipates, Sally's behavior by monitoring eye movements to assess the location of the moved ball. In another type of task tapping perspective-taking, researchers can evaluate the extent to which self-perspective-taking, such as determining the number of dots in a room, is influenced by the perspective of a task irrelevant agent, such as determining the number of dots from the perspective of an avatar.

Additionally, during all types of cognitive empathy tasks, affective or cognitive judgments can vary in their level of complexity, depending upon the number of “minds” (i.e., different individuals/agents) the participant needs to represent and track. For example, a first-order judgment is when an individual evaluates another agent's thoughts or feelings, only requiring that the individual represent one other agent's feelings or thoughts (e.g., evaluate if Character A likes Object X). A second-order judgment, however, is when an individual judges what another agent thinks about a third agent's thoughts or feelings, requiring the individual to simultaneously represent two other agent's feelings or thoughts (e.g., evaluate if Character A thinks Character B likes object X).

At a neurobiological level, cognitive empathy relies on the dynamic integration of information between a variety of cortical structures (50). Specifically, the medial prefrontal cortex, precuneus, and right temporoparietal junction are implicated in an individual's ability to judge another agent's feelings, beliefs, goals, or intentions (51–53). These regions appear to be common areas across subcomponent processes of cognitive empathy. Additionally, affective perspective-taking/ToM tends to elicit additional neural activation in the orbitofrontal cortex, ventromedial prefrontal cortex, amygdala, and superior temporal gyrus. Some research suggests that the amygdala acts as a detector when there are demands placed on affective perspective-taking/ToM through the presence of emotional or social stimuli (54). Cognitive perspective-taking/ToM may uniquely activate dorso-medial/lateral prefrontal regions (55).

Cognitive empathy allows individuals to recognize, understand, and predict how other agents will respond in social situations. These social cognitive processes provide a foundation for the formation and maintenance of social relationships that are mutually constructive. Researchers, clinicians, and lay people, alike, often note that those who engage in antisocial behavior lack cognitive empathy. But, what does the research actually tell us about the association between different subtypes of antisocial individuals and subcomponents of cognitive empathy?

## Cognitive Empathy in the Psychopathic Subtype

Across several studies, questionnaire-based evaluations of cognitive empathy reveal that higher levels of psychopathic/CU traits relates to lower levels of cognitive empathy (56–60). However, a closer examination of the research suggests that a more mixed pattern emerges depending on the informant (i.e., youth themselves vs. parent vs. teacher) and how these traits are modeled. These factors are especially important for youth samples. For example, when the individual in question was the informant, there was no relationship to small negative effects in the relationship between expressions of the psychopathic subtype and cognitive empathy, whereas the strongest negative relationships between this subtype and cognitive empathy were present when the questionnaires were completed by other informants, such as parents and teachers (61). Additionally, when CU traits were measured by themselves, reductions in questionnaire-measured cognitive empathy were apparent [e.g., (62, 63)]. By contrast, when CU traits were examined in the context of CD (e.g., CD+CU), there typically were no differences reported in cognitive empathy [e.g., (64)]. Thus, in terms of questionnaire-based assessments of cognitive empathy, the presence of deficits in the psychopathic subtype might be most observed by other informants or in those who have interpersonal-affective deficits but not necessarily conduct problems.

Research using behavioral tasks shows a divergence between cognitive and affective subcomponents of cognitive empathy. Across studies, neither youth with CU nor adults with psychopathy showed neural differences or behavioral deficits in cognitive ToM, suggesting intact cognitive ToM in psychopathy (65–79). By contrast, the evidence regarding the relationship between affective perspective-taking/ToM and psychopathy is more mixed.

To date, some studies reported that individuals with psychopathy were able to successfully assess another agent's affective state during affective perspective-taking/ToM tasks (70, 71, 73, 74, 76, 79), suggesting that individuals in the psychopathic subtype did not display deficits in affective perspective-taking/ToM. Conversely, other studies reported psychopathy-related behavioral abnormalities during affective perspective-taking/ToM tasks (64, 77, 78, 80, 81). For example, Sharp and Vanwoerden (78) demonstrated that, after viewing a 15-min long video clip depicting a dinner party (the Movie for the Assessment of Social Cognition task), adolescents high on CU were significantly worse than adolescents low on CU at evaluating what the characters in the film were feeling. Additionally, Shamay-Tsoory et al. (77) showed that after viewing a static cartoon image, adults with psychopathy were able to successfully make simple, first-order affective evaluations (e.g., Character A loves X object), but exhibited difficulty completing more complex, second-order affective evaluations (e.g., Character A loves the same object that Character B loves).

At first glance, these two studies appear to contradict the studies suggesting that individuals in psychopathic subtype show intact affective perspective-taking/ToM. However, it is possible that these apparently contradictory findings were actually the result of differences in task complexity. For example, Sharp and Vanwoerden (78) used a video of a dinner party as their task stimulus, requiring participants to process and track various pieces of information over the 15-min duration of the video. By contrast, other studies used relatively simple, static cartoon images, requiring participants to process and track, at most, three frames of information [(73, 76, 77, 79); see Roberts et al. (75) for a similar effect in cognitive ToM]. Similarly, Shamay-Tsoory et al. (77) reported that psychopathy-related difficulties in affective ToM were limited to complex, second-order judgments, which were not examined in any of the other studies. Collectively, these findings suggest that individuals in the psychopathic subtype exhibit difficulty with affective perspective-taking/ToM, but only when evaluating affective information that is embedded in a particularly complex stimulus (e.g., a movie), or when the judgment itself is highly complex or multilayered (e.g., second-order affective evaluations). This pattern of results suggests that when presented with more complex stimuli or scenarios, either the complexity of the scenario, the complexity of the affective judgments, and/or the amount of information required to process and track, impairs psychopathic individuals' ability to successfully evaluate or predict other agents' affective state.

Neural examinations of affective ToM in the psychopathic subtype yield similarly mixed results. On the one hand, several studies report that youth with CU traits or adults with psychopathy do not show substantial deficits during affective perspective-taking/ToM tasks (73, 80, 82). On the other hand, both Sebastian et al. (76) and Sommer et al. (79) reported that while individuals with psychopathy were able to successfully perform an affective ToM task (i.e., psychopathic individuals showed no behavioral differences compared to controls), they exhibited distinct neural abnormalities while performing the task. Sebastian et al. (76) specifically found that adolescents with CD who were high on CU (CD+CU) showed blunted amygdala responses during an affective ToM task that required participants to view and evaluate a static cartoon image. However, in their analysis, Sebastian et al. (76) examined amygdala reactivity across entire trials (i.e., during the initial presentation of the image and the judgment). This type of analysis made it difficult to determine what precise component of the trial was driving the blunted amygdala reactivity in adolescents with CD+CU. It is possible that the CD+CU-related blunting of the amygdala response was driven by neural differences when these youth initially saw (and affectively responded to) the cartoon images, rather than any CD+CU-related neural abnormalities in affective ToM (judgment).

Sommer et al. (79) reported that, during an affective ToM task, adults with psychopathy showed blunted responses in cortical regions associated with action observation and execution [i.e., the bilateral supramarginal gyri and superior frontal gyrus; (83)] and heightened responses in cortical regions generally associated with socio-affective processing, such as the orbitofrontal cortex, temporoparietal junction, and medial prefrontal cortex (51–53). This finding suggests that while adults with psychopathy were able to engage in affective ToM, they required more socio-affective neural resources to do so (79). While speculative, this need for additional neural resources to complete relatively simple (i.e., first-order) affective ToM judgments could potentially explain psychopathic individuals' apparent difficulties with more complex (i.e., second-order) affective evaluations (77). More specifically, psychopathic individuals may be able to engage enough neurocognitive resources to compensate for psychopathy-related difficulties in affective ToM during relatively simple, first-order, affective ToM evaluations. However, the additional neural resources needed to compensate for affective ToM deficits during more complex, second-order, affective evaluations may exceed the available neurocognitive resources for psychopathic individuals.

To this point, the studies reviewed exclusively examine tasks that explicitly instruct participants to engage in cognitive empathy, whether it is cognitive perspective-taking/ToM or affective perspective-taking/ToM. These studies do not assess whether individuals in the psychopathic subtype spontaneously engage in empathy (i.e., they have not assessed whether these individuals implicitly evaluate other agents' feelings, beliefs, goals, or intentions, in the absence of explicit instruction to do so).

A recent study by Drayton et al. (84) helped address this gap in the literature by examining the impact of psychopathy on an implicit measure of cognitive perspective-taking in an incarcerated sample. In this study, Drayton et al. (84) had inmates complete a cognitive perspective-taking task (49). During this task, participants were presented with static scenes depicting a gender- and race-matched avatar in a room with varying numbers of dots on the walls. The dots appeared in front of the avatar (i.e., the avatar had complete information), behind the avatar (i.e., the avatar had no information), or both (i.e., the avatar had partial information); however, the participant always saw all of the dots on every trial (i.e., the participant always had complete information). On some trials, participants were asked to evaluate how many dots the avatar could see (other-trials), and on some trials, participants were asked to evaluate how many dots they personally could see (self-trials). The other-trials provided a measure of explicit perspective-taking: could the participant take the avatar's perspective? The self-trials provided a measure of implicit perspective-taking: was the participant's perspective affected by the avatar's perspective? Research using this paradigm in the general population shows that when the avatar's perspective is different than the participant's perspective, participants are slower at reporting their own perspective (self-trials), indicating that individuals spontaneously take the avatar's perspective even if it is goal-irrelevant. Consistent with previous research on the psychopathic subtype, incarcerated individuals higher on psychopathy were able to engage in explicit perspective-taking and performed similarly to incarcerated individuals lower on psychopathy on the other-trials. However, incarcerated individuals higher on psychopathy compared to incarcerated individuals lower on psychopathy displayed significantly less interference on the self-trials (i.e., their reaction time was not affected by the perspective of the avatar). These findings suggest that psychopathic individuals do not implicitly evaluate others' mental states [i.e., they do not implicitly engage cognitive perspective-taking (84)], but can do so explicitly [see (75) for evidence of explicit abilities in CD+CU youth].

Another study examining pain perception in psychopathy suggests a similar pattern of psychopathy-related impairment in implicit affective ToM. Meffert et al. (85) used fMRI to examine neural responses to hand pain in three different conditions: passive viewing of a clip of a hand being hurt (i.e., implicit affective ToM), imagining what the person in the clip might be experiencing (i.e., explicit affective ToM), and physically experiencing the actual scenarios depicted in the clips. Meffert et al. (85) reported that, when adults with psychopathy passively viewed the pain clips, they did not exhibit significant neural overlap with their actual experience of pain (relative to controls), which the authors interpreted as evidence that adults with psychopathy did not implicitly engage in affective ToM. In contrast, however, Meffert et al. found that individuals with psychopathy showed similar overlap in neural responses to controls when instructed to imagine what the person was feeling (i.e., explicit affective ToM) and when physically experiencing the pain. These two findings suggest that adults with psychopathy are able to engage in affective ToM, but do not do so implicitly (i.e., without instruction).

While the purely neural nature of these findings makes this interpretation somewhat speculative, these findings and interpretations are consistent with both prior research demonstrating psychopathy-related neural abnormalities in pain perception in others (86), and other findings indicating that individuals with the psychopathic subtype do not implicitly engage in cognitive perspective-taking (75, 84). Thus, the current literature examining cognitive empathy in the psychopathic subtype provides strong evidence that individuals in this subtype largely are able to engage in cognitive empathy when instructed to do so, but do not do so implicitly. This is an important distinction because it helps in explaining why individuals in the psychopathic subtype can so easily manipulate others' thoughts and feelings when conning them (as the act of conning someone explicitly requires empathy), yet have difficulty with more everyday social interactions, which may require more implicit empathy. While social interactions in the real-world are inherently more complex than experimental tasks that have a participant watch a dinner party or view an avatar, the deliberate instruction during tasks, or explicit goal-focus in the real-world, may alleviate some of the processing burden that undermines empathetic functioning in individuals within the psychopathic subtype.

**Figure 2:**
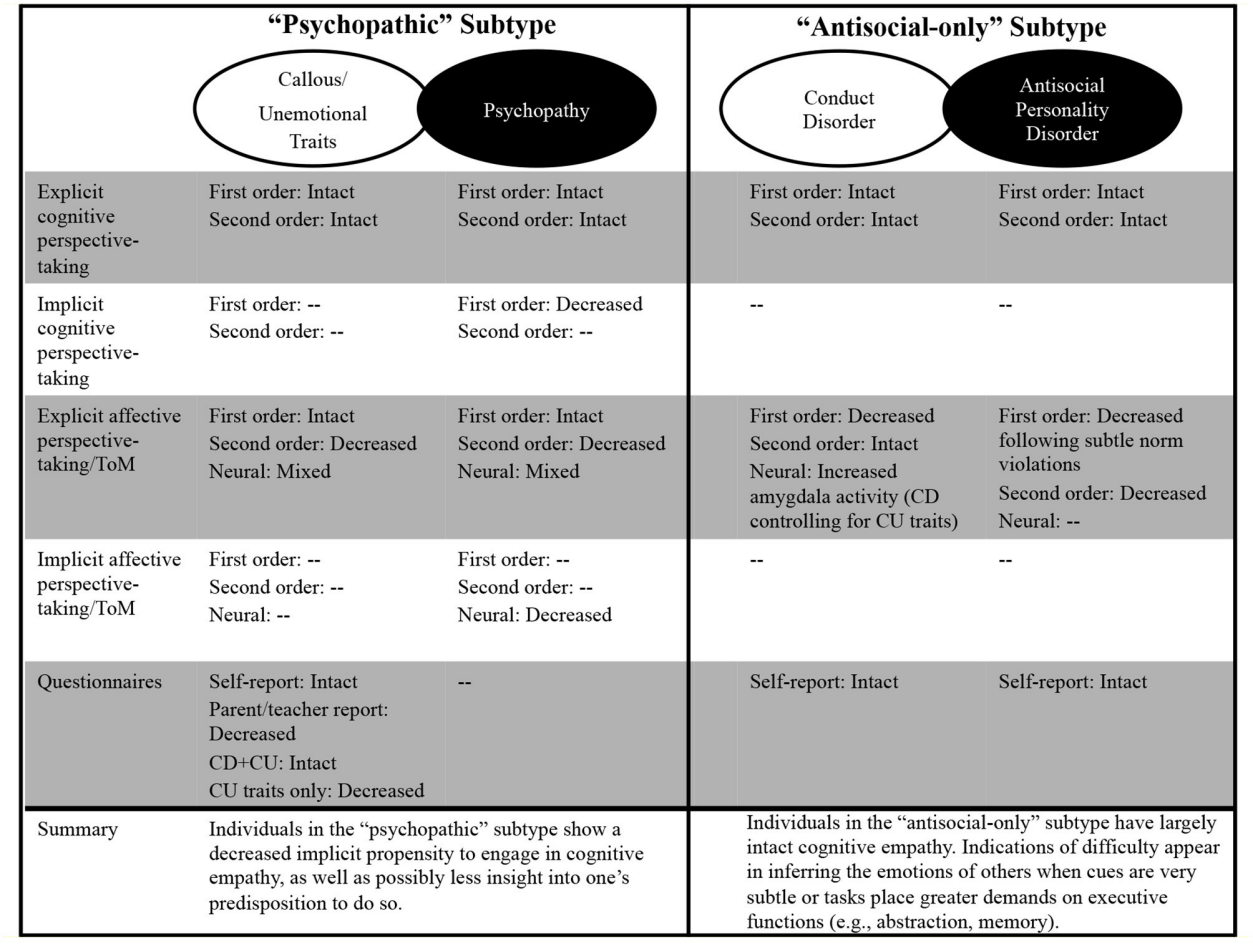
Summary of cognitive empathy findings by subtype of antisocial individual. CU, callous-unemotional; CD, conduct disorder; ToM, theory of mind; --, indicates no research to date.

Overall, research indicates that individuals in the psychopathic subtype may not have a complete deficit in cognitive empathy (see Figure 2). When individuals in psychopathic subtype are asked to report on their own empathy or complete simple, cognitive empathy, tasks, empathy appears intact. However, when other observers are asked to report on the behavior of CU youth, or psychopathic individuals are asked to engage cognitive empathy in more complex situations, deficits are more apparent. Moreover, a recurrent finding across various aspects of cognitive empathy in psychopathy is that, even if individuals within this subtype can normatively engage different empathetic processes (in specific circumstances), they tend to only do so when instructed. The failure to implicitly attend to, and process, others' emotions or mental states, combined perhaps with a lack of self-awareness about this tendency, may explain how these individuals are able to callously harm others during goal-pursuit, but also able to charm, con, and manipulate others when necessary.

## Cognitive Empathy in the Antisocial-Only Subtype

For the antisocial-only subtype, questionnaire-based evaluations of cognitive empathy suggested that these processes are intact (70, 87). Similarly, when assessed behaviorally, several studies demonstrated that individuals in the antisocial-only subtype, across all developmental stages, exhibit intact cognitive empathy (68, 75, 76, 88–90). Though, admittedly examination of cognitive empathy in the antisocial-only subtype has been less systematic than research in the psychopathic subtype. For example, no studies have examined implicit cognitive empathy in antisocial-only individuals. Despite the overall pattern of intact cognitive empathy in the antisocial-only subtype, some research indicates that the specific demands of the tasks reveal nuanced dysfunction in subcomponent processes of cognitive empathy.

Across youth and adult samples, individuals in the antisocial-only subtype (CD; ASPD) display dysfunction when there is a specific demand on affective perspective-taking/ToM (64, 87, 91, 92). For example, Dolan and Fullam (66) reported that, while individuals with ASPD were able to successfully complete traditional false belief tasks and identify subtle violations of social norms (e.g., identify when someone accidentally said something that should not have been said; i.e., social faux pas), they exhibited difficulties with affective ToM within the context of these subtle norm violations. More specifically, adults with ASPD displayed difficulties assessing characters' affective states/perspective after the characters experienced a subtle norm violation. In another study, Newbury-Helps et al. (91) administered several cognitive empathy tasks in a sample of justice-involved individuals. Individuals with ASPD displayed a particularly pronounced deficit in affective ToM during the Movie for the Assessment of Social Cognition task, scoring in a range that reflected difficulty with memory, general comprehension, and abstraction.

Examination of neural differences in cognitive empathy for individuals in the antisocial-only subtype has been limited. Sebastian et al. (76) reported that, during an affective ToM fMRI paradigm assessing second-order judgments, CD symptomology (controlling for CU traits) in adolescents was unrelated to behavioral task performance. However, CD symptomology was associated with increased amygdala reactivity across the entire trial to affective vs. cognitive ToM scenarios after controlling for CU traits (76). The effect of increased amygdala activation in this study could be the result of neural abnormalities in affective ToM, or simply the product of increased amygdala reactivity when initially seeing (and affectively responding to) the affectively valenced scenes. Regardless of the specific interpretation, however, at a neural level there may be evidence that antisocial-only individuals, especially compared to individuals in the psychopathic subtype, over-react to affective information (see (93, 94) for similar effects in inferring the pain of others [i.e., first-order judgment]).

Generally, research examining cognitive empathy in the antisocial-only subtype shows that these individuals exhibit intact cognitive empathy when measured through questionnaires and behavioral tasks that tap explicit empathic functioning (see Figure 2). Antisocial-only individuals appear to attend to, recognize, and make inferences about social cues. However, individuals in the antisocial-only subtype may display some difficulty inferring the emotions of others. Though research has been limited in this subtype, it is possible that evidence of some affective ToM dysfunctions reflects issues with executive functions, such as abstract reasoning, and imprecision in detecting and regulating affective capacities (37, 76, 95–97). Antisocial-only individuals tend to display deficits in executive functions, such as flexibility and abstract reasoning (98). These executive functions are necessary for a full range of empathetic functioning, including picking up on subtle affective cues. Moreover, problems with executive functioning, combined with dysfunction in affective processing that reflects over-and-under-responding in various situations (99), can undermine regulated responding to affective information. Thus, impairments in inferring the emotions of others when the signals are subtle and difficulty remembering or comprehending the emotions of others may result in the unpredictable, perhaps impulsive, interpersonal interactions characteristic of these individuals. Moreover, possible over-reactivity to salient affective information may generate an explosive, poorly regulated, reaction from antisocial-only individuals in these interpersonal contexts.

## Considerations for Future Research and Conclusions

There is both clinical and empirical support for cognitive empathy disruptions in psychopathic and antisocial-only subtypes of individuals. However, a close examination of the available data suggests that the specific manner of dysfunction varies between subtypes of individuals and subcomponents of cognitive empathy. Individuals within the psychopathic subtype appear to be viewed by others as deficient in cognitive empathy (based on questionnaires), but show adequate performance on cognitive empathy tasks, particularly when explicitly asked to engage empathy. However, in cognitive and affective perspective-taking/ToM tasks, these individuals appear not to engage these processes automatically, requiring instructions to direct their attention to relevant socio-affective information in order to respond normatively. Antisocial-only individuals reliably report intact cognitive empathy and are able to perform reasonably well on behavioral tasks that tap explicit processes, but may struggle to fully comprehend or process affective signals, particularly if subtle. Overall, differences in cognitive empathetic functioning differentiate these two subtypes of individuals and may relate to their differential phenotypic expressions.

The specific nature of the problems with cognitive empathy in psychopathic and antisocial-only individuals further highlight the equifinality of antisocial behavior. Despite both subtypes of individuals exhibiting chronic violations of social norms and a disregard for others, the processes underlying their behavior appear distinct. Moreover, the pattern of dysfunction in cognitive empathy for these subtypes of individuals follows a larger literature on cognitive-affective functioning in psychopathic and antisocial-only individuals. On the whole, cognitive empathy dysfunction in the psychopathic subtype, particularly their ability to explicitly engage cognitive empathy but their deficient propensity to implicitly do so, may echo the broader cognitive-affective deficits these individuals have in attending to and integrating multiple streams of information (38, 100). Similarly, individuals in the antisocial-only subtype do not appear to engage in antisocial behaviors because of a fundamental deficit in cognitive empathy. In fact, the situations when dysfunction in cognitive empathy are apparent (e.g., inaccurate affective perspective-taking following subtle violations, over-reacting neurally during affective ToM) may reflect cognitive-affective dysfunctions related to deficits in executive functioning and poor affective regulatory capacities that just happen to arise during cognitive empathy tasks, which place demands on these functions (11, 37). Noting the consistency in dysfunction across levels of analysis is important for future work that may explore the specific processes that underlie complex social cognitive dysfunction in antisocial individuals.

The identification of subtype-specific core cognitive-affective dysfunctions that cut across levels of analysis raises an important question of whether the cognitive-affective dysfunctions lead to the psychopathic or antisocial-only expressions or are just related to these expressions. Very few longitudinal studies examining the development of antisociality and cognitive empathy have been conducted. In one study, displays of concern for others, which encompasses a range of affective and cognitive indicators of empathy, at age 14 to 36 months, did not predict ASPD at age 23 years. However, observed disregard for others, which represents responding to other's distress with anger or hostility, predicted the interpersonal-affective traits of psychopathy, and ASPD (101). In another study, cognitive ToM at 4.5 years old did not predict CU traits at 10 years old (102). Cognitive ToM did predict impulsive behavior at 10 years old, but this relationship was better accounted for by exhibition of externalizing behaviors (conduct problems, hyperactivity) at age 5. Thus, very preliminary evidence suggests that cognitive empathy does not predict antisociality, and, that affective sensitivities may be more likely as candidate processes that pre-date antisociality (101, 102). Far more research examining subcomponents of empathy that span cognitive and affective domains is needed.

The type of measure selected to tap cognitive empathy within each antisocial subtype also reveals interesting divergences. Notably, within and across antisocial subtypes, there were inconsistencies depending on whether questionnaires or tasks were used, and even depending on the specific task being used. For the differences between questionnaires and task performance, these inconsistencies may reflect the fact that questionnaire-based measures often fail to precisely capture a specific process of cognitive empathy, whereas many tasks are more specifically designed to tap a subcomponent of cognitive empathy. Thus, researchers must accurately label and discuss the measures being used in their particular study. Moreover, specific biases in certain tasks may lead to deficit performance but may not actually reflect a deficit in cognitive empathy *per se*. For example, the Movie for the Assessment of Social Cognition uses a white middle-class dinner party as a key stimulus. Cognitive empathy is sensitive to in-group and out-group effects (103, 104), as such, participants who are not white and middle-class may have difficulty identifying with the characters. Thus, any performance deficits on this task may not be because of a failure to represent the characters' thoughts and feelings, but rather an unfamiliarity or disconnect with the experiences presented in these clips due to larger sociocultural differences. Therefore, researchers should consider ways to match stimuli and participant characteristics [see (84) for example], and to develop more culturally sensitive measures of cognitive empathy.

Another aspect of cognitive empathy that requires further exploration is the distinction between explicit and implicit cognitive empathy. The handful of studies in the psychopathic subtype highlight the value in distinguishing between implicit and explicit engagement of cognitive empathy, underscoring that individuals in the psychopathic subtype lack the propensity to implicitly engage cognitive empathy but not the ability to explicitly engage cognitive empathy (75, 84, 85). The distinction between implicit and explicit empathy also may be reflected in the questionnaires dissociations observed in youth with CU traits. It is possible that youth endorse cognitive empathy on a questionnaire (i.e., show an explicit ability to recognize the appropriate response), but do not engage with it naturally or implicitly in the day-to-day life witnessed by others. Research within the antisocial-only subtype has not compared implicit vs. explicit tendencies in cognitive empathy. Disentangling whether someone has an ability to explicitly engage cognitive empathy vs. lacks a propensity to implicitly to do so has important clinical implications. The presence of an ability to explicitly engage cognitive empathy, but the absence of an implicit propensity, suggests that compensatory strategies that allow antisocial individuals to circumvent their cognitive-affective deficits (e.g., difficulty processing and tracking complex stimuli) may be beneficial for increasing prosocial behavior. For example, by instructing individuals with psychopathy or CU traits to focus on key social information (e.g., facial affect, contextual cues about the situation), these individuals may be able to more deliberately integrate this information. While empathy itself may not be normalized, the behavior of those with psychopathy or CU traits has the potential to reflect the use of important social information by making the focus on that information more deliberate.

Beyond specific processes supporting cognitive empathy in antisocial subtypes, little research in this domain accounts for the contribution of environmental risk factors that are related to both the quality of cognitive empathy functioning and subtype of antisociality. For example, early childhood deprivation, maltreatment, and poverty occur at high rates among individuals who chronically engage in antisocial behavior (105, 106). Outside of research on antisociality, early childhood maltreatment and other environmental factors, such as concentrated disadvantage, are known to negatively impact empathetic functioning and development (107–109). For example, children who are maltreated experience substantial deficits and delays in ToM (107, 109). Accordingly, it is possible that some of the deficits associated with antisocial subtypes are promoted by certain environmental experiences. However, research examining the intersection of antisociality, early environment, and cognitive empathy is limited, making this possibility hard to evaluate, but an exciting endeavor for future research.

The relationship between cognitive empathy and antisociality is complex. Lay beliefs that antisocial individuals must engage in antisocial behavior because they are incapable of cognitive empathy are not supported by extant literature. Rather, dysfunction in cognitive empathy appears dependent on subtype of individuals and subcomponent process of cognitive empathy. Advancing our understanding of the links between cognitive empathy disruptions and antisocial subtypes is crucial to providing unique insight into the development and maintenance of the chronic, disruptive, and costly behaviors exhibited by these individuals.

## Author Contributions

SAC and AB-S wrote the first draft of the manuscript. ST wrote sections of the manuscript. All authors contributed to manuscript revision, read, and approved the submitted version.

## Conflict of Interest

The authors declare that the research was conducted in the absence of any commercial or financial relationships that could be construed as a potential conflict of interest.

## Footnotes

^1^This review is a part of a special topic examining “Cognitive Empathy and Perspective Taking: Understanding the Mechanisms of Normal and Abnormal Experiences and Abilities.” Thus, we focus our discussion on cognitive empathy, as opposed to affective empathy, which refers to the ability to emotionally resonate with or experientially share another person's internal affective state.
